# Safety and effectiveness evaluation of open reanastomosis for obliterative or recalcitrant anastomotic stricture after radical retropubic prostatectomy

**DOI:** 10.1590/S1677-5538.IBJU.2017.0681

**Published:** 2019-04-01

**Authors:** Carlos Roberto Giúdice, Patricio Esteban Lodi, Ana Milena Olivares, Ignacio Pablo Tobia, Gabriel Andrés Favre

**Affiliations:** 1 Department of Urology, Reconstructive Surgery Area, Hospital Italiano de Buenos Aires, Argentina

**Keywords:** Erectile Dysfunction, Prostatectomy, Urinary Incontinence

## Abstract

**Purpose:**

To evaluate safety, efficacy and functional outcomes after open vesicourethral re - anastomosis using different approaches based on previous urinary continence.

**Materials and Methods:**

Retrospective study of patients treated from 2002 to 2017 due to vesicourethral anastomosis stricture (VUAS) post radical prostatectomy (RP) who failed endoscopic treatment with at least 3 months of follow-up. Continent and incontinent patients post RP were assigned to abdominal (AA) or perineal approach (PA), respectively. Demographic and perioperative variables were registered. Follow-up was completed with clinical interview, uroflowmetry and cystoscopy every 4 months. Success was defined as asymptomatic patients with urethral lumen that allows a 14 French flexible cystoscope.

**Results:**

Twenty patients underwent open re-anastomosis for VUAS after RP between 2002 and 2017. Mean age was 63.7 years (standard deviation 1.4) and median follow-up was 10 months (range 3 – 112). The approach distribution was PA 10 patients (50%) and AA 10 patients (50%). The mean surgery time and median hospital time were 246.2 ± 35.8 minutes and 4 days (range 2 – 10), respectively with no differences between approaches. No significant complication rate was found. Three patients in the AA group had gait disorder with favorable evolution and no sequels.

Estimated 2 years primary success rate was 80%. After primary procedures 89.9% remained stenosis - free. All PA patients remained incontinent, and 90% AA remained continent during follow-up.

**Conclusion:**

Open vesicourethral re - anastomosis treatment is a reasonable treatment option for recurrent VUAS after RP. All patients with perineal approach remained incontinent while incontinence rate in abdominal approach was rather low.

## INTRODUCTION

Radical prostatectomy (RP) is a well - established procedure for the treatment of localized prostate cancer (1-4).

Most frequent long term complications mentioned are: sexual dysfunction, urinary incontinence and vesicourethral anastomosis stenosis (VUAS). The latter one, is a rare but troublesome complication with an incidence of approximately 8.4% (5-10). It's well known that the endoscopic approach provides good results; success rates vary from 50 to 91% with a mean of 2.1 interventions per patient (2, 11, 12).

Despite this, some patients show VUAS recurrence after endoscopic approach, in which case an open surgical reconstruction is the recommended procedure.

Open procedures can be addressed by different approaches: perineal, abdomino - perineal and abdominal (12-14). Perineal approach has the advantage of being an unspoiled surgical access, nevertheless, because of the urethral mobilization, this approach is associated with high rate of urinary incontinence (UI) (11, 15), thus, an artificial sphincter urinary (AUS) is mandatory. Some authors recommend that all patients must be counselled that this will almost certainly be a two - stage reconstruction, the first to clear the urethral obstruction by revision of the vesicourethral anastomosis and the second to implant an artificial sphincter for the almost inevitable sphincter weakness incontinence following this clearance (16).

Since the VUAS is proximal to the sphincter, some authors prefer the abdominal approach in order to preserve the external sphincter function and therefore the continence. Other advantage may be to keep the bulbar urethra intact in case there is a need for a subsequent AUS implantation.

This paper presents an update of our experience in open re-anastomosis for recurrent VUAS by either perineal or abdominal approach.

## OBJECTIVES

To evaluate safety, efficacy and functional outcomes after open vesicourethral re - anastomosis (ORA) using different approaches based on previous urinary continence.

## MATERIALS AND METHODS

Retrospective observational study. Data from patients treated for VUAS post radical prostatectomy in our hospital from 2002 to 2017 was retrospectively analyzed. The data collection was prospectively done from the electronic clinical history.

Patients with recalcitrant VUAS post RP (defined as the failure of more than three endoscopic treatments) and those with obliterated VUAS post RP were included in the analysis. Patient were included in this study only if they had at least 3 months of follow-up.

All patients underwent preoperative retrograde urethrography and voiding cystourethrography. Approach was chosen according to continence status before open reconstruction. We define as continent, after radical prostatectomy and subsequent endoscopic attempts for VUAS, as no need of any pads or only one. Incontinence was defined as the need of more than one pad. For continent patients, abdominal approach was chosen in order to preserve external sphincter. Perineal approach was offered to incontinent patients considering the benefit of an undamaged surgical field.

Variables registered were: surgical time, need for blood transfusion, intraoperative complications, hospital convalescent time, as well as postoperative complications related to the different approaches (complications were assessed using the Clavien - Dindo score), orthopedic complications / issues, and free rate re-stenosis and postoperative UI defined as the use of more than one pad per day.

Follow-up was carried out with clinical interview, uroflowmetry and cystoscopy every 4 months. Success was defined as asymptomatic patients with urethral lumen that allows a 14 French flexible cystoscope. Failure was defined as the need for any new treatment in order to restore the urethral lumen after ORA.

Continence after ORA was defined by the need of pads: one or none as continent and more than one as incontinent. Erectile dysfunction was defined as the patient's inability to achieve an erection that allows penetration.

For this study we inform the results of the last follow-up or those at the time of re - stenosis to avoid self - correlation bias.

## SURGICAL PROCEDURES

Perineal approach: With the patient in a forced lithotomy position, lambda perineal incision was made, dissection of planes to reach the VUAS. Flexible cystoscopy (14 Fr) was done to confirm localization of the stenosis. Extensive mobilization of the anterior urethra was performed. Opening of the crura and / or partial pubectomy was performed if needed. In patients with patent urethral lumen, an urethral catheter was introduced with cystoscopic aid. In cases with complete obliterated stenosis an abbocath^®^ catheter was introduced into the bladder through the fibrotic tissue guided under cystoscopy by the suprapubic traject. With this maneuver we perform the anastomosis in the anatomical bladder neck spot.

**Figure 1:**
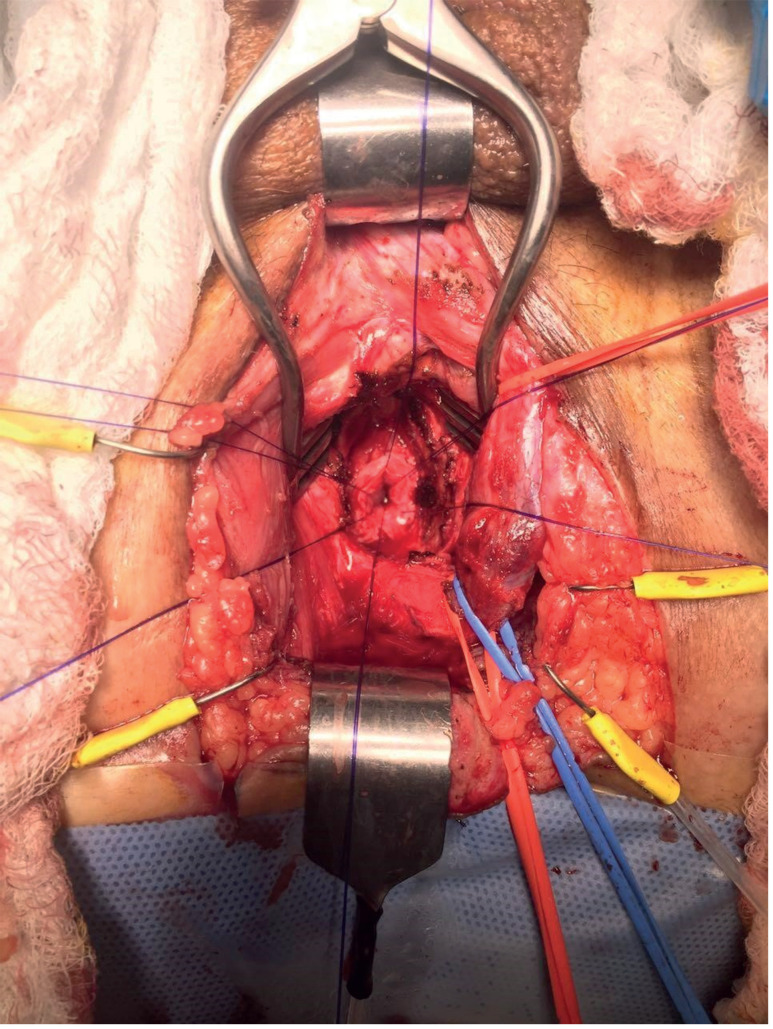
Urethral lumen previous to re anastomosis by perineal approach. Note that the bulb was not transected so proximal irrigation is intact.

Resection of the scarred tissue segment and vesicourethral re - anastomosis was constructed with six interrupted sutures of PDS 4 / 0. When possible, the corpus spongiosum was not transected (Figure-1).

**Figure 2:**
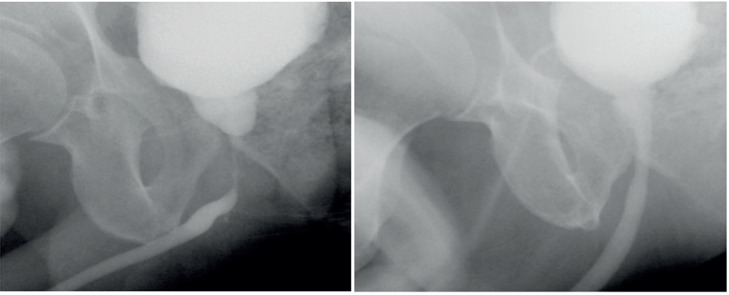
Pre and post ORA cystourethrography in perineal approach.

Silastic 18 Fr urethral catheter was placed, which was removed under radioscopic control after 3 – 4 weeks (Figure-2).

**Figure 3:**
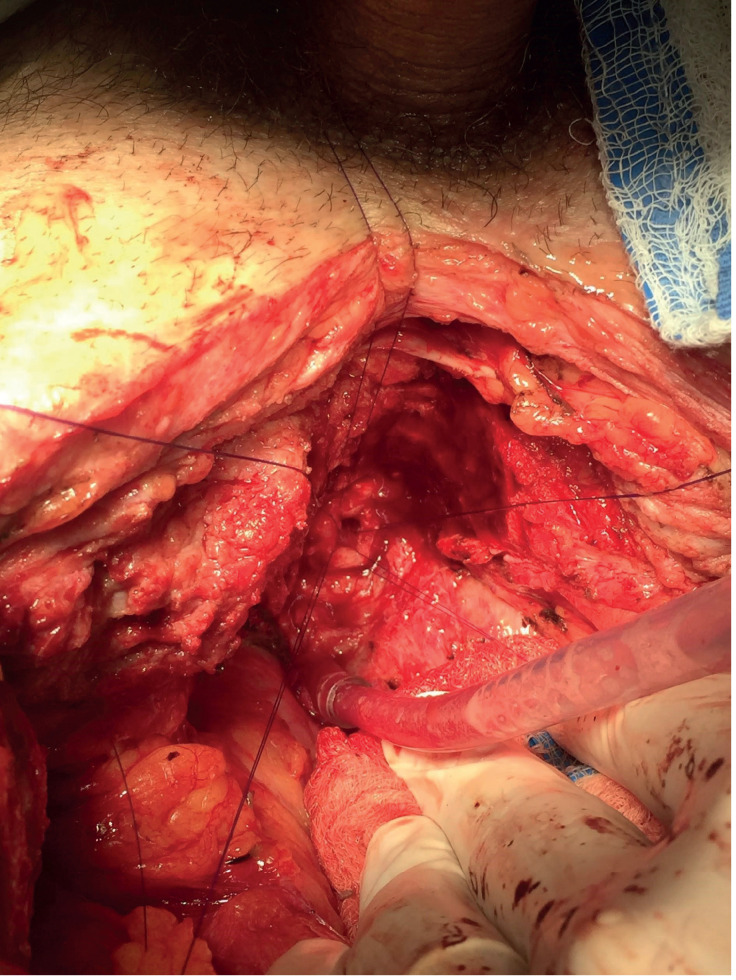
Urethral lumen previous to re anastomosis by abdominal approach. Note that the pubectomy provide a comfortable surgical access to the stricture area.

**Figure 4:**
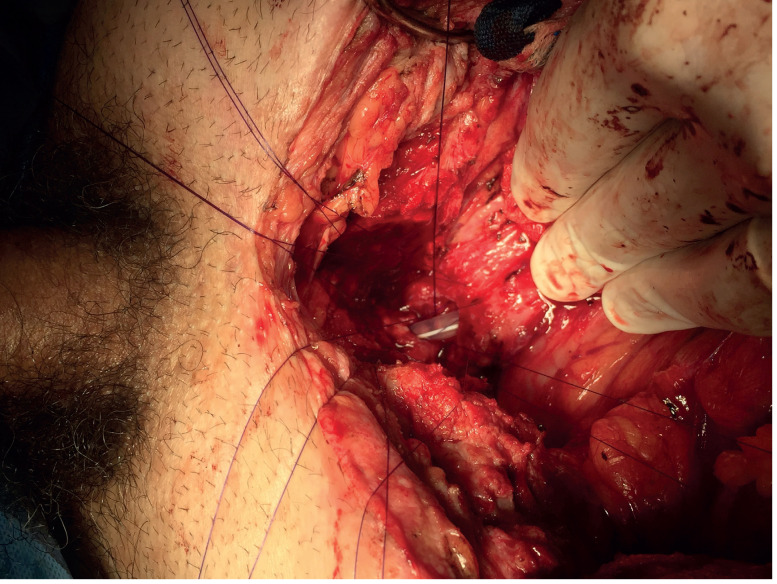
Reanastomosis by abdominal approach.

**Figure 5:**
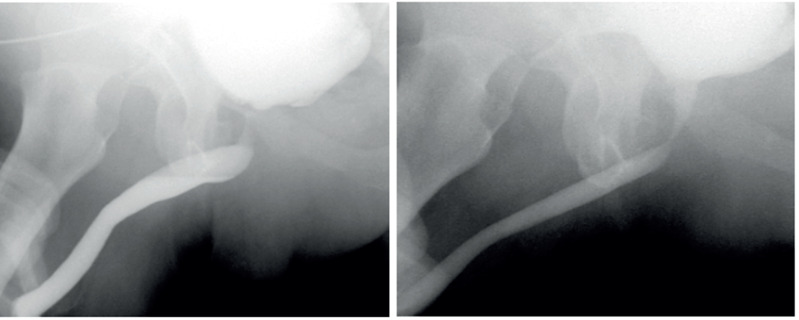
Pre and post ORA cystourethrography in abdominal approach.

Abdominal approach: The patient was placed in dorsal decubitus, infraumbilical medial incision was made, dissection of the pre - vesical area was performed. After complete mobilization of the bladder was achieved, partial pubectomy was performed to access the vesicourethral anastomosis site. The stenotic site was identified with a flexible cystoscope (14 Fr) and at the point of the stenosis, the bladder neck is divided. The fibrosis is removed and healthy bladder is dissected from the rectum. With benique^®^ catheter through the urethra, placed in retrograde fashion, the urethra is dissected around the benique^®^ and the fibrosis is completely removed. Urethral and bladder mobilization is necessary to achieve a tension free anastomosis. Then, we performed re - anastomosis with PDS 4 / 0 interrupted sutures (Figures 3 and 4). Silastic 18 Fr catheter and suprapubic cystostomy were placed. The urethral catheter was removed under radioscopic control after 3 – 4 weeks (Figure-5).

Statistical analysis: continuous variables with normal distribution are informed as their mean and standard deviation (sd). If there is non - parametric distribution, they are expressed by their median and range (r). For comparison, t test or Mann Whitney are utilized. Categorical variables are expressed as their value and percentage (%). For their comparison, Fisher exact test is employed. For survival estimation, Kaplan Meier method was chosen. In all cases, a p value < 0.05 is considered with statistical significance. The software utilized was SPSS 21.0 (™).

## RESULTS

Twenty patients underwent open re - anastomosis for VUAS after RP between July 2002 and June 2017. Demographic data is described in Table-1.

**Table 1:** Demographic data.

Mean age years (sd)		63.7 (1.4)
**Type of surgery (%):**		
	Radical prostatectomy	13 (65)
	Laparoscopic radical prostatectomy	4 (20)
	Salvage radical prostatectomy (post radiotherapy)	3 (15)
	Adjuvant radiotherapy (%)	5 (25)
**Comorbidities (%):**		
	Diabetes	2 (10)
	Obesity	3 (15)
	Smokers	4 (20)
Mean number of endoscopic treatments post RP (sd)		2.26 (1.8)

The median follow-up after ORA was 10 months (r 3 – 112).

The approach distribution was: perineal 10 patients (50%) and abdominal 10 (50%). The mean surgery time was 246.2 ± 35.8 minutes with no differences between approaches (perineal 248.9 ± 69; abdominal 229.5 ± 22.1, p 0.61). No significant intraoperative complications were recorded, no rectal or ureteral orifices injuries were evidenced and no patient required blood transfusion. Post operatory data is described in Table-2. Minor postoperative complications were similar in both groups. Length of hospital stay was higher in the abdominal approach group, where gait disorders were exclusively present. This gait disturbance is fully associated with partial pubectomy. Patients referred during the first 20 to 30 days, limp due to pelvic bone pain, needing help from a cane, with a spontaneous resolution within 30 days after surgery, only requiring nonsteroidal anti - inflammatory drugs (NSAIDs) orally.

**Table 2:** Post operatory data.

	Overall (n=20)	Perineal (n=10)	Abdominal (n=10)	p
Median hospital convalescent time, days (r)	4 (r 2-10)	3 (2-4)	4 (3-10)	0.03
Postoperative complications (%)	7 (35)	2 (20)	5 (50)	0.35
Clavien- Dindo I	3 (15)	1 (10)	2 (20)	
Clavien- Dindo II	4 (20)	1 (10)	3 (33.3)	
Disorders in the gait (%)	4 (20)	0	4 (40)	0.07

### Success rate

The estimated 2 years primary success rate was 80% (95% IC 62.6 – 97.4). Median time to primary recurrence was 6 months (r 1 – 36). Of the 6 recurrences, 4 were in the perineal approach group and 2 in the abdominal approach group (p 0.329). Two of this six patients were irradiated patients (one in each group). Median follow-up time after primary procedure was 19.5 months (r 3 – 106). All recurrences were treated with one minimally invasive procedure (5 patients internal urethrotomy and 1 urethral dilatation). Only 9 patients had a follow-up longer than 24 months, in this population, after minimally invasive procedures, overall success rate was 89.9%.

Median follow-up after secondary procedure was 24 months (r – 12 – 108). During follow-up, 19 patients (95%) achieved mean Qmax of 19 mL / sg (r 13 – 32 mL / seg).

### Urinary incontinence

All patients that underwent perineal approach were completely incontinent following re - anastomosis, and were treated with anti - incontinence devices or are scheduled for treatment. Three patients in the abdominal approach developed “de novo” urinary incontinence (p 0.003). One of these patients presented severe UI and was treated with AUS, this patients belonged to the radiated group. The other two underwent biofeedback therapy due to their mild UI, one of them with good response. After this treatment, 9 of 10 patients were continent (90%) (Table-3).

**Table 3:** UI treatment and evolution by approach.

Treatment	Perineal approach (Evolution)	Abdominal approach (Evolution)
Artificial Urinary Sphincter	2 patients (1 extrusion: required a new AUS)	1 patient (Actually continent)
Sling	2 patients (Actually continent)	0
Biofeedback	1 patient (Good response:1 pad/day)	2 patients (1 Good response:1 pad/day) (1 Not response:3or more pads/day)
No treatment by the time the data was analyzed	5 patients (2 planning Anti-incontinence device)	0

### Erectile dysfunction

As regards erectile dysfunction, 19 patients presented this affection after RP. Only one patient had normal erectile function post RP, and this condition was maintained after ORA.

## DISCUSSION

Vesicourethral stenosis after radical prostatectomy is an uncommon and difficult complication to treat. Literature analysis, in some cases with an antiquity greater than 10 years, describes an incidence that varies from 0.5 to 32 % (2, 3, 5-9, 17). VUAS etiology is not yet clear; inadequate contact mucosa - mucosa appears to be the genesis of this complication and most important risks factors related are smoke habits, radiotherapy, obesity, previous TURP, surgeon unexperienced, hematoma and urinary leak (5, 7, 9, 13).

Endoscopic management in non - obliterative VUAS after RP appears to be the first option. Controversy exists regarding which endoscopic approach is better. Recently, LaBossiere et al., compared the results obtained with different endoscopic modalities treatment for VUAS and report that holmium laser incision appears to have more success compared to other modalities (2). Some authors suggest the use of intralesional antiproliferative substances improves outcomes (11, 13, 18). Endoscopic approach in obliterated VUAS is not only non - effective but also unsafe (18, 19).

Despite these results, approximately 10% of the patients will not respond to endoscopic treatments (2). In these patients, the options frequently considered are urinary diversion, suprapubic cystostomy and open re-anastomosis. This last procedure is reserved for healthy and well - motivated patients and has the advantage of preserving the bladder with the intrinsic benefits.

ORA can be accomplished by perineal, abdominal and abdominal / perineal approach (12, 13).

Perineal approach offers the advantage of being free of previous surgeries with unscarred tissue, however the most important problem is the trans - sphincteric mobilization of the urethra and consequent UI. Recently, Cavalcanti et al. described a series of 48 patients with VUAS addressed by perineal approach. Twenty four of them (50%) presented UI (20). In addition, Ivaz et al., stated that all patients must be counselled that this will almost certainly be a two - stage reconstruction, the first to unblock them by revision of the vesicourethral anastomosis and then secondly to implant an artificial urinary sphincter for the almost inevitable sphincter weakness incontinence following unobstruction (16). This is supported by our data, where all 10 patients that underwent perineal approach were incontinent following re - anastomosis and the majority of them were offered to receive an anti - incontinence treatment.

AUS is considered the gold standard for the treatment of UI after VUAS re - anastomosis by perineal approach. Despite the utility of the AUS, it is well known the association with complications and urinary incontinence post implantation vary between 12 and 40% (20-25). Recently, successful implantation (17 / 23 patients) has been reported with AUS for UI after perineal approach (15). In the majority of their patients the authors describe a double cuff was implanted and 4 cases needed revision or explantation. On the other hand, Nicolavky and colleges, reported that AUS cuff erosion occur only in patients with previous urethral mobilization by perineal approach (26). In our series, 2 patients in the perineal group, were implanted with a AUS, with one of them suffering cuff erosion.

Considering the VUAS is proximal to the sphincter, the abdominal approach would allow the re - anastomosis to be performed leaving the external sphincter intact and thus the patient's continence. Abdominal approach is considered more complex, since the need of an aggressive bladder mobilization and, in some cases, a wide pubectomy in a previous scarred surgical field. We do not report significant differences between approaches regarding surgery time, need of blood transfusion or minor postoperative complications. Length of hospital stay was higher in abdominal approach group, where gait disorders were exclusively present. As regards to this last complication, patients refer the first 20 to 30 days limp due to pelvic bone pain, with a spontaneous resolution within 30 days after surgery. Patients only required oral NSAIDs as analgesic. This complication is fully associated with pubectomy. Complete removal or incision of the pubis will adequately expose the posterior urethra and distal bladder neck but the stability of the pubis may be compromised. Literature describes children that suffered from chronic pain and gait disturbances after this procedure (27). Although gait disorders have full recovery, patients must always be advised before surgery if abdominal approach is chosen.

Even Wessels et al. (28) present a series with 100% of UI after ORA by abdominal approach; most recently Pfalzgraf et al. reported a 64% preserved continence after ORA with this approach (14), prevalence that seems to be similar to our series, where 7 of 10 (70%) patients that underwent abdominal approach preserved their urinary continence. In our report, in the 3 patients who developed de novo UI, just one required an AUS because he presented severe UI. The other two underwent biofeedback therapy due to their mild UI. After this treatment, 9 of 10 patients were continent (90%).

Overall, our stenosis free rate of ORA in the treatment of recalcitrant VUAS after RP is 89.9% despite the approach with a median follow-up of 10 months (3 – 112). These results are similar to the ones reported in the literature, where different approaches achieved good results (12). When we look at the 9 patients with global follow-up more than 2 years, 4 (44.4%) were treated with minimally invasive procedures (median time 16.5 months, 2 abdominal and 2 perineal approach). In this patients success rate after minimally invasive treatment was 89.9% with a median follow-up after that treatment of 19.5 months.

We reported a set of complications that are different depending of the approach. In the abdominal group, the more frequent complication was related with the pubectomy. Four patients referred disorders in the gait for at least a month after surgery, with complete recovery after that period of time. We do not have clear explanation of these complications other than the stability of the pubis may be compromised after pubectomy. Another complication related to this approach is the presence of fistula (1 patient), event not observed in patients who underwent perineal approach.

This paper has some limitations. Due to the low prevalence of this kind of pathology, the number included is low, so conclusions could not be so strong. Follow-up median time was 10 months, with half of patients with less than one year of follow-up, which is too short for a cohort study. This short follow-up may lead us to bias because overestimation of success rate, even when we inform success rate of the sample of patients with follow-up larger than 2 years. Follow-up after minimally invasive treatment may be too short to establish real success rate which is the problem of this rare pathology.

On the other hand, we consider our report as a novel task. There are few published papers about this issue on Latin American patients. In the translational medicine era, having publications of this sort of pathologies is a big help for urologists to know how to deal with them.

## CONCLUSIONS

Open treatment of vesicourethral anastomosis has overall success rate of 89.9% despite the approach. All patients with perineal approach remained incontinent. On the other hand, abdominal approach presents an incontinence prevalence of 10%. No major complications were observed in any procedure. After abdominal approach, gait disorders may occur with complete recovery achieved in a month as average time.
